# How social policies can improve financial accessibility of healthcare: a multi-level analysis of unmet medical need in European countries

**DOI:** 10.1186/s12939-016-0335-7

**Published:** 2016-03-05

**Authors:** Sabine Israel

**Affiliations:** Faculty I–School of Educational and Social Sciences, Carl von Ossietzky University Oldenburg, Ammerlaender Heerstrasse 114-118, 26129 Oldenburg, Germany

**Keywords:** Unmet medical needs, Access to healthcare, Great Recession, Social expenditure, EU SILC

## Abstract

**Background:**

The article explores in how far financial accessibility of healthcare (FAH) is restricted for low-income groups and identifies social protection policies that can supplement health policies in guaranteeing universal access to healthcare. The article is aimed to advance the literature on comparative European social epidemiology by focussing on income-related barriers of healthcare take-up.

**Method:**

The research is carried out on the basis of multi-level cross-sectional analyses using 2012 EU-SILC data for 30 European countries. The social policy data stems from EU-SILC beneficiary information.

**Results:**

It is argued that unmet medical needs are a reality for many individuals within Europe – not only due to direct user fees but also due to indirect costs such as waiting time, travel costs, time not spent working. Moreover, low FAH affects not only the lowest income quintile but also the lower middle income class. The study observes that social allowance increases the purchasing power of both household types, thereby helping them to overcome financial barriers to healthcare uptake.

**Conclusion:**

Alongside healthcare system reform aimed at improving the pro-poor availability of healthcare facilities and financing, policies directed at improving FAH should aim at providing a minimum income base to the low-income quintile. Moreover, categorical policies should address households exposed to debt which form the key vulnerable group within the low-income classes.

## Background

Social protection policies complement public health policies in improving population health in two ways. Firstly, they reduce the risk of illness [1, 2] when they address the unequal distribution of detrimental social determinants (such as substandard living conditions) [3, 4]. Secondly, they ameliorate the chances of receiving necessary treatments and medical consultancies for the ill by increasing the disposable income of poor households, facilitating financial access to healthcare. While the healthcare system is often portrayed as the main point in order to address official hurdles to *healthcare access*, social policies can decrease the unofficial hurdles towards *healthcare take-up*, lowering enforced lack of healthcare due to income constraints.

Poor households^1^ belong to the groups that are most easily deterred from the take-up of healthcare services [5]. Yet also the lower middle income classes have shown increased difficulties in accessing healthcare in recent years [6, 7]. Disincentives to using healthcare services stem not only from direct user fees charged at a healthcare centre but also from indirect costs of the visit, such as money spent on transport or medication co-payments, as well as opportunity costs related to time spent out of work [8]. This paper will focus on individuals from the two lowest income quintiles reporting income-related absence of medical care using EU SILC data from the 2012 wave. This includes “enforced” unmet medical needs for reasons of waiting lists, transportation costs or costs of treatment. It will address the following questions: What is the importance of social protection policies next to the organisation of the healthcare system for access to healthcare? Can social cash benefit programs help low-income groups to overcome the (remaining) demand-side barriers to accessing healthcare? Are different barriers and policy solutions salient for the poorest quintile and those at the lower middle income class? The aim of the article is to advance the literature on comparative European social epidemiology by focussing on income-related problems of healthcare take-up. The contribution differs from previous research by setting the focus not on the supply side of medical services, but on the side of the individual whose demand is pre-structured by the policy context. It takes the patient-centred, integrated health system perspective [8–10], by looking at the various stages involved in the process of seeking care, and applies it to the European context.

The article is structured as follows: First, the concept of financial accessibility of healthcare (FAH) is clarified and its supply and demand-side factors explained. Subsequently, the article will turn to the risk factors of FAH for working-aged low-income groups in Europe. Using logistic multilevel analysis, the impact of social protection benefits on accessing healthcare is analysed and quintile-specific regressions are carried out. The article concludes with recommendations on the types of social protection programmes that can most effectively complement healthcare policies in improving FAH among the low-income groups.

### Defining access to healthcare

In this paper, the patients’ perspective on accessibility is adopted (see also [11, 12]), defining *access to healthcare* as “the timely use of service according to need” [13]. All EU countries guarantee (quasi) universal healthcare coverage for a basic service package by either universal, citizenship-based or insurance-based healthcare arrangements [14]. Nonetheless, such legal rights do not imply equal quality of care for all groups, nor do they remove all barriers to service take-up.

**Fig. 1:**
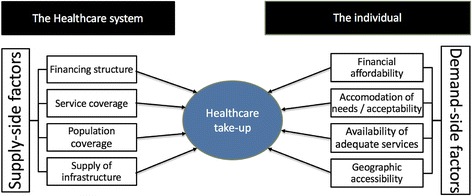
Demand- and supply-side determinants of healthcare take-up. Note: Own representation. Supply-side factors based on [28, 29, 11] Demand-side factors based on [15, 16]

Adequate accessibility depends not only on the characteristics of the healthcare system, but also on the action of an individual to access healthcare, such as taking the step towards addressing one’s health issues or searching for a preventive action (see Fig. 1). Penchansky and Thomas define access as “a measure of ‘fit’ between characteristics of providers and health services and characteristics and expectations of clients” [15]. They outline five dimensions describing the “demand-side” of healthcare access [15, 16]: 1) availability of adequate services, 2) geographical accessibility, 3) affordability, 4) accommodation of a patient’s needs, and 5) acceptability of services for patients. *Availability* measures factors such as confidence in receiving good medical care and knowledge of the care system; *accessibility* the convenience of getting to the physician’s office; *accommodation* the ease of contacting the physician and waiting times; *affordability* the satisfaction with health insurance, pricing and payment conditions; and *acceptability* the appearance of the doctor’s office. Levesque et al. deduce four corresponding abilities (the ability to seek, reach, pay and engage) and add the “ability to perceive an illness” as their fifth, predisposing factor [10].

Guaranteeing financial accessibility to low-income groups is not limited to the affordability of user charges. In addition to direct treatment costs, financial accessibility also encompasses indirect costs, as listed in the other four dimensions of access above. Risk factors linked to geographical accessibility, accommodation, and acceptability are thus interrelated with affordability and worsen when households are at the same time exposed to financial restrictions [10]. Travel costs and waiting time are clearly regarded as opportunity costs by patients, as shown by Penchansky and Thomas, and reduce patients’ satisfaction with the affordability of healthcare [15]. Moreover, a comprehensive concept of financial accessibility must imply that individuals are not only able to afford treatment but also that they are protected from impoverishment when faced with costs related to visiting a healthcare service [17]. An adequate definition of *financial accessibility of healthcare* (FAH) is therefore the timely use of services according to need *without the risk of impoverishment*.

### Household determinants of take-up of healthcare services

FAH is known to be a particular problem to individuals with low household resources. Economic power still plays a central role in access to healthcare in European countries. When comparing individuals with the same health-related needs, high-income households are more likely to contact a general practitioner in 15 EU countries (all part of the OECD), and more likely to contact a specialist in all OECD countries [18]. Mielck et al. also report higher amounts of unmet medical needs in low-income groups than in high-income groups in France, Germany, Greece, Italy and Sweden using the SHARE study [19]. Hart concluded that individuals with the highest need and vulnerability are those least likely to receive medical care, calling it the ‘inverse care law’ [20].

For certain groups of the population, FAH is more likely due to barriers in the four aforementioned dimensions of access. In insurance-based healthcare systems access to healthcare is linked to the employment status of an individual. In this case individuals who become unemployed, self-employed or inactive have to change their insurance status, which can lead to a loss of healthcare coverage [21, 22]. Non-coverage implies that individuals will need to pay the full costs of the services by themselves thus constituting a key barrier to healthcare use. Another important factor is an individuals’ knowledge of the healthcare system and of changing rules. Awareness of the system influences an individual’s ability to access healthcare through access points that are exempt from fees. For instance, in so-called gate-keeping systems, charges are waived for specialist visits if they are based on a referral from a GP [12]. Individuals with personal barriers in accessing information such as low education or language barriers can have problems of managing the healthcare system and thus decide not to take-up care [23]. Moreover, households in rural areas or deprived urban areas are particularly likely to report FAH for reasons related to geographical accessibility. In rural areas, the lack of public transport and required travel time can can be considered a financial disincentive [24]. In deprived urban areas, the offer of medical care often does not correspond to the increased need of poor and unemployed households and communities [25].

### Structural determinants of the take-up of healthcare services

The design of the ‘health-care state’ [26] is highly responsible for the decommodification of healthcare [12, 27]. Structural “supply-side” factors which determine FAH relate to the four basic dimensions of healthcare: 1) provision of infrastructure, 2) population coverage, 3) service coverage and 4) financing [11, 28, 29]. Provision of infrastructure constitute the basis of each country’s healthcare system. Infrastructure includes the number and distribution of general practitioners (GPs), specialists and hospitals, their staffing and equipment. Understaffed hospitals and large distances from medical care units will increase waiting times and travel costs, thereby reducing the accommodation and affordability of healthcare services for individuals. Population coverage for healthcare systems in Europe is close to universal [14]. Before the European growth, debt and unemployment crisis, 19 out of the 27 countries had achieved full coverage, whilst the other eight European countries (mostly welfare states with an insurance-based system) were covering around 95 % of the total population [28]. The coverage of services, on the other hand, refers to the publicly paid benefit package. The same service can be fully covered, partially covered or not be covered by different European health arrangements, thereby largely influencing the cost of care (for both individuals and the government).

With population coverage being close to universal, healthcare financing turns into the most important structural barrier to FAH. Financing refers to the private-public share of healthcare contributions. In general, one can state that the higher the private payments as a percentage of total health expenditure,^2^ the greater “the privatisation of health” in the case of sickness [30]. Private expenditures are made up of expenditures for diagnostics, pharmaceuticals and medical goods that are not included in the basic service package, and user fees as well as informal payments in certain countries [31]. User fees are private out-of-pocket payments (OPP), which the individual pays directly after the contact with healthcare services.^3^ As they consist of a lump sum instead of being distributed progressively throughout the income strata, they form a high burden on low-income households.

Recent healthcare policy reforms, which have been engendered by the Great Recession,^4^ have often resulted in shifting costs for healthcare to the individual, making the financing more regressive [7, 28, 32, 33]. In all European countries, however, the key vulnerable groups are benefiting from exemptions to payments or fee reductions. These groups are in most cases children, pregnant women and mothers with young children, elderly, low-income individuals, and individuals with chronic illnesses [31]. Nonetheless, even in rich European countries co-payments are creating financial barriers to access, delaying visits and reducing health service utilisation [5, 11, 18]. Even though co-payments are implemented to reduce visits with low-value, in reality they discourage low-value and high-value visits to the same extent [5].

### Social policies supporting FAH for different income groups

Households belonging to the first and second income quintiles struggle with various problems linked to FAH. In addition, these same households are also exposed to different social protection policies, i.e. compulsory governmental schemes which protect from risks or pool risks under the principle of social solidarity. In the following, it will be argued how such social protection policies can support health policies in improving FAH.

#### The risks of low-income groups

**Fig. 2:**
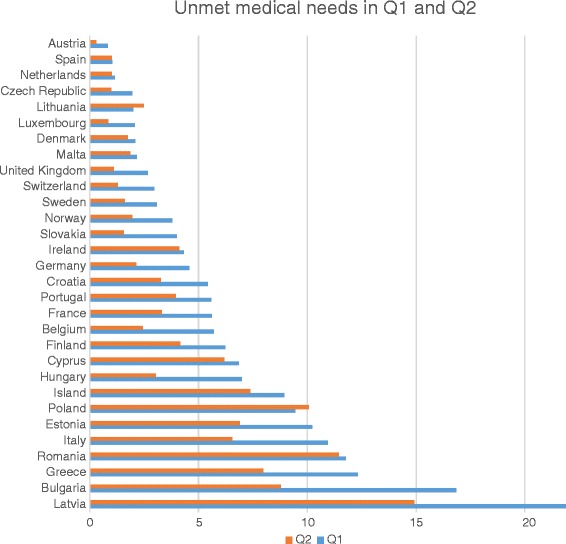
Comparing income-based unmet medical needs across quintiles, 2012 (in % of quintile). Note: EU SILC data, Q1 refers to the poorest income quintile, Q2 to the lower middle income quintile

When looking at the effect of social protection policies on FAH, it is important to distinguish between different income groups. For individuals belonging to the lower income quintile (Q1), user charges do not constitute the main barrier to service take-up. Indirect costs such as informal charges, transport and pharmaceutics costs can however lead to reduced affordability. The lower-middle class (Q2) does in general not benefit from exemptions. They proportionately bear the highest burden of lump-sum user charges. This disproportionality of direct costs increased following the Great Recession and the healthcare policy reforms put in place in most European countries.^5^ Consequently, the risk of low FAH for households from the lower middle classes has risen [7]. In Romania, Poland, Ireland and Lithuania the risk of the lower middle class is according to 2012 data as high or even higher than the risk of the lowest population quintile (see Fig. 2). *H1 assumes therefore that indirect costs such as transport availability and distance to healthcare centres will be among the most important risk factors for Q1, while household characteristics related to the financial power and to healthcare coverage (such as unemployment and debt issues) will be more relevant for Q2*.

**Fig. 3:**
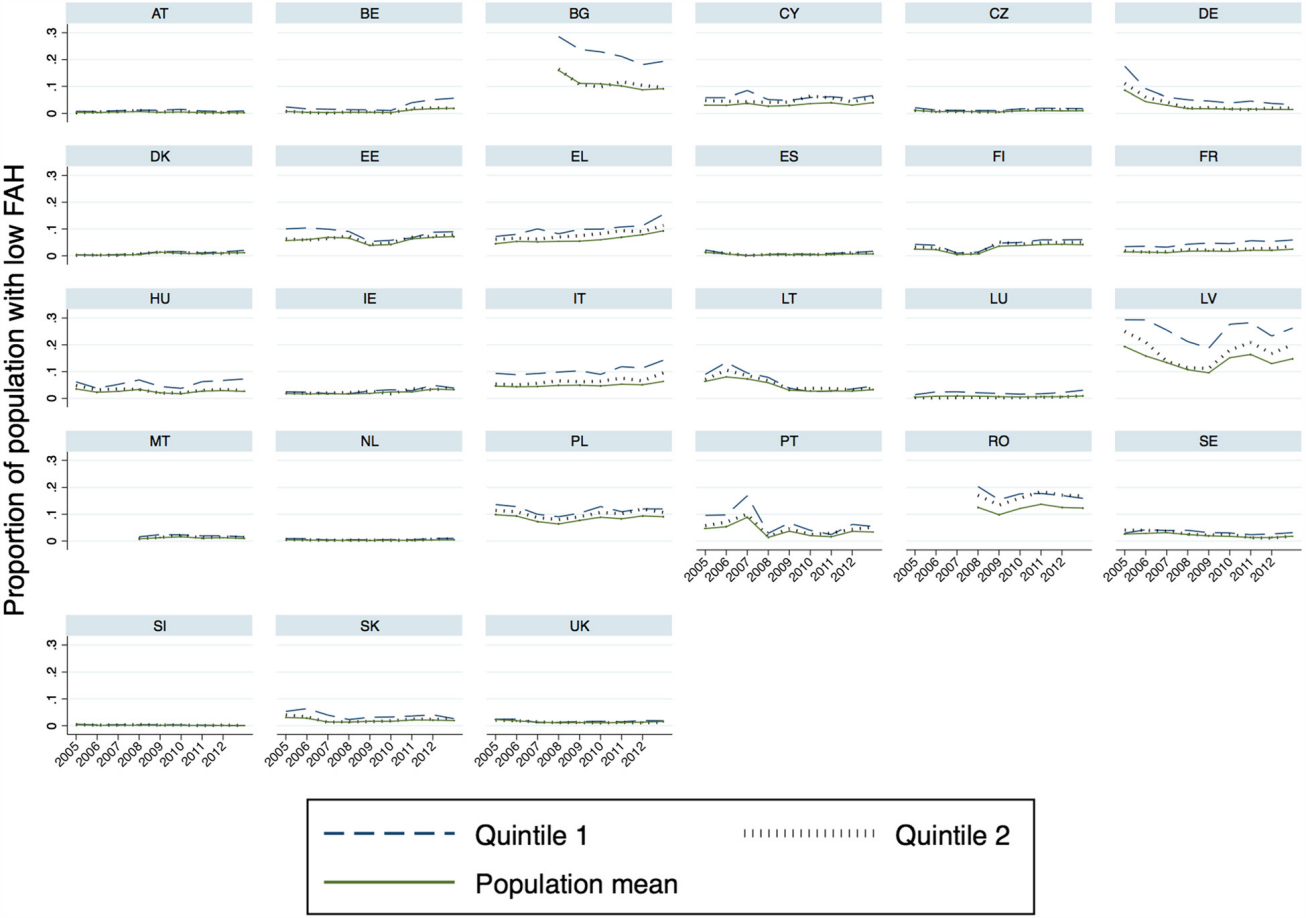
Income-based barriers to healthcare access for different quintiles in EU countries. Note: EU SILC data. Timeline constructed with cross-sectional time-series data (TCSC). Q1 refers to the poorest income quintile, Q2 to the lower middle income quintile

#### Relevance of social protection next to healthcare policies

Previous studies, analysing the mechanisms that mitigate household’s risk of low FAH, portrayed mainly measures to be carried out from within the healthcare sector. Gelormino et al. [34] provides the broadest approach towards addressing the inequality in access to care for the European Union [34]. They propose four main points, including more progressive financing, socially-selective allocation of health resources to places with the highest need prevails, positive discrimination in the regulation of health demand towards the poor (including reduced waiting lists and co-payments for this most vulnerable group), as well as a higher equity-orientation in care-giving. More recently, Eurofound identified a need for implementing adequate formal entitlements to care, informing individuals about their rights in the healthcare system and scaling up screening and measures to address urgent needs [7]. While these *healthcare system* approaches are decisive for improving the supply of healthcare, the broader *social protection policies* in which the individual is embedded determine the demand-side barriers to healthcare access. For the ultimate decision of taking up healthcare, the interaction between the person’s characteristics and those of the healthcare system play a key role. Looking at FAH, direct and indirect costs form the highest hurdle towards taking-up healthcare in Europe. Thus, it is expected that that social policies, particularly those increasing household disposable income, facilitate healthcare take-up. *H2 is assuming that expenditure on social protection programmes improve FAH*.

#### Quintile-specific policies

Social protection policies that improve the purchasing power of households from the lowest income quintile are minimum income programmes, such as social allowance and housing benefits [35] (see Table 1). Social allowance refers to means-tested benefits available for households which fall into certain categories (e.g. single parents) or which qualify through their low household income [36]. It functions as a safety net with the goal of providing households with enough disposable income to master their minimum living costs.^6^ On average, this benefit is topping up household income of the lowest income quintile in the EU with an equivalised 281€ (see Table 1). Housing benefits in a form of rent reimbursements or public housing provision are likewise often part of anti-poverty programmes. EU SILC data shows that its eligibility is more selective than that of social assistance, targeting selectively the very poor. Throughout the EU, on average 194€ of equivalised housing benefits are paid to the income poor. H3a therefore assumes that *social policies geared towards improving the purchasing power of low-income households such as social assistance and housing benefits improve these households’ FAH*.

**Table 1:** Social allowances (per month) and their targeting of poor population parts

Social protection benefits	Mean (in PPP)	Mean Q1 (in PPP)	Mean Q2 (in PPP)	Min/Max (in PPP)
Family allowance	506€	92€	104€	47/1643€
Social allowance	101€	281€	90€	0/387€
Unemployment allowance	390€	42€	75€	35/1601€
Housing allowance	132€	194€	102€	0/581€

Note: calculated using 2012 EU SILC data

Different social protection elements, such as unemployment benefits and family benefits (either earnings-related or universal) increase the purchasing power of the lower-middle class. These are households with incomes above the poverty line, which are at risk of getting into a ‘poverty trap’ (i.e. not being able to exist unfavourable living conditions) when high user charges are placed on them. Family benefits (made up of child benefits, tax allowances and family allowances) vary greatly in amount and criteria across European countries. In most EU countries, family benefits are universal, meaning that they are not dependent on the household income [37]. EU SILC data shows that the allowance paid to Q1 and Q2 is similar, with Q1 receiving 104€ and Q2 92€ per child (see Table 1). The earnings-related unemployment benefits, on the contrary, are paid as a percentage of former earnings (replacement rate), clearly benefiting the lower-middle income group by topping up their household income with 75€ on average, double of that received by the lowest income group. Following H3b assumes that *categorical and income-related benefits (*e.g. *family benefits, unemployment benefits) increase the purchasing power of households from lower-middle income group and improve their FAH*.

## Methods

The study is based on multi-level cross-sectional analyses of EU SILC 2012 data, the official statistics on income and living conditions of the European Union. EU SILC provides detailed information on all EU28 Member States plus Norway, Iceland and Switzerland. The data collection methods vary depending on country between administrative records, national registers and household interviews between countries, as the EU-SILC is output harmonized [38]. All countries are included in the analysis, except for Croatia and Cyprus due to missing data for the macro-level variables. The unit of analysis is the individual. However the sample is restricted to working-age persons (18–65 years), as different policies might apply to the retired. In total, we count 283,078 cases from 30 countries.

### Conceptualisation of FAH and independent variables

Access to healthcare is defined as unmet medical needs, which arise if a person would have liked to contact a doctor but had restricted access to medical diagnosis and treatment. In EU SILC, “unmet need for medical examination or treatment” (ph040) is analysed using two questions. First, the individual is asked if during the last 12 months, there was any occasion on which he or she really needed to consult a medical doctor but did not. Second, different reasons can be given for the unmet need for medical examination. Under the definition of FAH (Financial accessibility of healthcare) adopted in this paper, persons stating the following reasons for an unmet medical need were included: waiting lists, transportation costs, and costs of treatment (including non-coverage by medical insurance). In this way individuals with multiple and overlapping risk factors are captured in the definition of FAH. The individual-level characteristics included in the regression analysis control for general health, chronic illness, sex, and household composition as variables that influence the perception of need (see Table 2). Migrant status and education are also included in the model as variables capturing the knowledge of the healthcare system. Finally, urbanisation, accessibility of public transport and accessibility of primary care control for the impact of indirect costs, and basic activity status, income before taxes and benefits, and debt (eviction due to financial reasons) control for the impact of direct costs. The country-level variables concerning social protection were obtained by aggregating EU-SILC data. While the beneficiary data has the advantage of showing group differences, it comes at the expense of being gross data. Therefore, robustness checks with expenditure data will be carried out to confirm the validity of the results. The control variables regarding out-of-pocket expenditure and physician density were retrieved from the WHO and OECD databases, respectively. Before including them into the models, macro variables were z-standardized and divided by 100 to increase the effect size.

**Table 2:** Operationalisation, data sources and the expected effects of variables

Variable	Operationalisation	Expected effect
*Dependent*		
Limited FAH (unmet medical needs due to financial reasons)	Unmet need for medical examination/treatment (ph040) for reasons of (ph050): costs (1), transportation costs (4) or waiting lists (2) (0: no income-related need; 1: income-related need)	
*Independent*		
Bad health	Subjective health (0: (very) good; 1: average/(very) bad (ph010)	+
Chronic illness	Suffer from any chronic (long-standing) illness or condition (ph020)	+
Sex	1: male (ref. group); 2: female (rb090)	+
Household composition	1: ‘Adults, no children’	(ref.)
	2: ‘Adults with children’	+
	3: ‘Single parent household’ (hx060)	+
Migrant status	Non-/EU migrant by country of birth (pb210) or citizenship (pb220)	+
Education	1: ‘Primary education’ ISCED 0–2 (pe040)	+
	2: ‘Secondary education’ ISCED 2–4	+
	3: Tertiary education’ ISCED 4–6	(ref.)
Urbanisation	1: densely populated,	(ref.)
	2: intermediate area,	+
	3: thinly populated area (db100)	+
Access to public transport	From 1 (With great difficulty) to 4 (very easily) (hc120)	+
Access to primary healthcare services	From 1 (With great difficulty) to 4 (very easily) (hc130)	+
Basic activity status	0: at work (ref. group), 1: unemployed (rb210)	+
Debt problems	Household will be forced to leave the dwelling (hc150)	+
Income (pre-tax/benefit)	Gross equivalised disposable income in PPP (log) (hx090)	−
*Macro-level*		
Housing allowance	Gross equivalised housing allowance, including rent benefit, benefit to owner-occupiers (hy070g)	−
Social assistance	Allowance for social exclusion not elsewhere classified, including income support and cash benefit for vulnerable groups (hy060g)	−
Family allowance	Gross equivalised family allowance, including birth grant, parental leave benefit, family or child allowance, gov. alimonies (hy050g)	−
Unemployment benefits	Gross individual unemployment benefits, including full/partial unemployment, early retirement and severance benefit (py090g)	−
Physician density	Density of practising and professionally active physicians per 1000 population (head counts) (source: OECD)	−
Out-of-pocket payments	Out-of pocket expenditure/Total health expenditure (source: WHO)	+

Note: The expected effect shows the hypothesised effect of the dependent variables on unmet medical needs due to financial reasons. + implies a positive correlation (linked to an increased risk of unmet needs), while – implies a negative correlation (linked to a reduced risk of unmet needs). *(ref.)* indicates the reference group for categorical variables

### Multi-level methodology

The probability of being exposed to low FAH i.e., the probability of experiencing unmet medical needs due to financial reasons, is calculated using multi-level models. These are applied as EU-SILC data has a hierarchical structure, meaning that observations are nested within a higher-level unit (in this case individuals within nation-states). Hierarchical data requires a special regression type as responses from within one country tend to be more similar, so that correlations between error terms may arise [39]. This paper applies the simple 2-level variance components model in a logit form, which is constructed with the Stata command for binary outcome analysis xtmelogit:

$$ {y}_{ij}={\beta}_0+{\beta}_1{x}_{ij}+{\beta}_2{z}_j+{u}_j+{e}_{ij} $$

In the equation, ‘yij’ refers to the event that household ‘i’ in country ‘j’ has no access to medical care for cost reasons. ‘β0’, the intercept, denotes the average risk of low FAH for households independent of the country they are located in. ‘xij’ is an individual-level explanatory variable, ‘zj’ a macro-level explanatory variable and ‘β1’ and ‘β2’ the corresponding coefficients. ‘eij’ and ‘uj’ decompose the error variance with ‘eij’ referring to the individual level error and, ‘uj’ to the national level error [40].

The following models are estimated: The empty model (M0) forms the basis to estimate goodness of fit measures. Subsequently, M1 includes individual-level variables and controls for health policy on the macro level. In the next models, social policy variables are added stepwise. M2 displays the full model and tests H*2*, which assumes that expenditure on social protection programmes improve FAH. M3 and M4 split the full model for the first and second quintile respectively. By calculating the risks for Q1 and Q2 separately, the analysis enables distinguishing risk factors and policies that are particularly harmful or helpful for the two lowest income groups. In this way, the general trend of averaging the risks of various different population groups is opposed, which is a method that may mask particularly vulnerable groups [7, 41]. Comparing M3 and M4 on the micro level, H1 is assessed, which stated that indirect costs (transport and distance to healthcare) will be a key determinant of FAH for Q1, while financial power (unemployment and debt) will be more relevant for Q2. When comparing M3 and M4 on the macro-level, H3a and H3b are evaluated which expected housing and social assistance to be relevant social protection factors for Q1, and family and unemployment benefits to influence the FAH of Q2.

Multi-level models have been criticised to provide unstable higher-level estimates when covering few countries. Comparing Monte-Carlo simulations of non-linear multi-level regression for EU-SILC, Bryan and Jenkins estimate that with a minimum number of 30 cases, reliable results are obtainable [42]. This analysis follows the advice by using 30 countries and by applying ML with adaptive Gaussian quadrature as the estimator, which has been shown to produce accurate estimates [43]. The obtained results seem robust. Even when Bonferroni correction for multiple testing [44] are applied, which give highly conservative *p*-value estimates, no substantial changes in the results occur.

The coefficients displayed are average marginal effects (AME). AMEs average the result of discrete or partial changes in the coefficient for x over all observations [45]. They have two specific advantages over odds ratios when applied in logit models [46, 47]: Firstly, AMEs are not affected by unobserved heterogeneity, i.e. omitted variables. Secondly, AMEs can be more easily compared across population groups. As AMEs are additive approximations of effects, they can be interpreted as percentage point differences (an AME of 0.05 corresponds to a 5 percentage point increase).

## Results

The most common reason for an unmet medical need in 2012 was the cost of medical care (36 %), followed by waiting list, which deterred help seeking behaviour (15 %). Transportation was an issue for only around 3 % of those stating an unmet medical need. In total, problems of low FAH thus constituted a bit more than half of all access problems in 2012 and concerned a proportion of 3 % of the European population. While this percentage may seem low, a high amount of variation exists across the EU and a fluctuation becomes apparent over time (see Fig 3). Most European countries seem to be able to keep their promise of quasi universal access to healthcare (in particular AT, CZ, DK, ES, MT, NL, SI, UK), but in some countries more than 10 % of the population is hindered. Latvia, Romania, Poland and Estonia have the highest percentage of people with low FAH. As shown, the probability for the poor is still higher, at 22 % in Latvia, 17 % in Bulgaria, 14 % in Romania and around 11 % in Italy, Greece and Poland. Figure 2 also shows that until 2009 an amelioration in the proportion of people with limited FAH took place (in particular in CEEC countries). From 2010 onwards, however, a renewed worsening of access to healthcare took place, which affected the lowest income quintile most in Greece, Spain, Ireland and Hungary.

### Multilevel analysis: the public policies behind FAH in Europe

In Table 3, the multilevel analysis predicting the probability to experience low FAH (measured as unmet medical needs due to financial reasons) is displayed for different population groups while holding the degree of bad health and chronic illness constant. First, the individual characteristics leading to financial difficulties in accessing medical services are laid out for the whole population, then for the quintiles. As a second step, the macro-level influences are discussed for the total model and specifically for Q1 and Q2.

**Table 3:** Individual and country-level determinants of income-related unmet medical needs

Category	Variable	M1	M2	M3	M4
Sample Population		ALL	ALL	Q1	Q2
Health	Bad health	0.035**	0.037**	0.062**	0.050**
		(8.43)	(9.28)	(9.42)	(8.95)
	Chronic illness	0.010**	0.011**	0.018**	0.013**
		(8.07)	(8.81)	(7.66)	(7.59)
Sex (Ref. male)	Women	0.007**	0.007**	0.010**	0.010**
		(6.81)	(7.24)	(4.41)	(5.22)
Household composition (Ref. 2 adults, no child)	Single parent	0.008**	0.009**	0.008+/o	0.008*/o
		(4.70)	(4.85)	(1.88)	(2.10)
	Household with children	0.000	0.000	−0.001	−0.002
		(0.26)	(0.26)	(−0.56)	(−1.05)
Migrant status (Ref. national)	Migrant	0.005**	0.005**	0.012**	0.002
		(4.07)	(4.19)	(3.53)	(0.87)
Education (Ref. Higher education)	Low education	0.009**	0.009**	0.015**	0.005+/o
		(6.30)	(6.11)	(3.63)	(1.76)
	Medium education	0.002**	0.003**	0.006	−0.002
		(2.97)	(3.00)	(1.56)	(−0.70)
Location	Access to primary care	−0.008**	−0.008**	−0.012**	−0.009**
		(−7.69)	(−7.40)	(−6.29)	(−5.80)
	Access to public transport	−0.000	−0.000	−0.003*/o	−0.001
		(−0.52)	(−0.07)	(−2.25)	(−1.16)
Employment (Ref. Employed)	Unemployed	0.015**	0.017**	0.019**	0.017**
		(7.19)	(7.09)	(5.22)	(4.47)
Debts	Eviction for financial reasons	0.024**	0.025**	0.042**	0.044**
		(6.44)	(6.80)	(5.34)	(6.06)
Financial situation	Gross income (in log)	−0.008**	−0.008**	−0.007**	−0.007**
		(−8.23)	(−9.02)	(−6.57)	(−5.06)
Health policy					
Financing	Out-of-pocket expenditure	0.077*/+	0.063+/o	0.001+/o	0.120**
		(2.08)	(1.81)	(1.83)	(2.66)
Infrastructure	Physician density	−0.012*	−0.013**	−0.020*	−0.015*
		(−2.50)	(−2.87)	(−2.55)	(−2.64)
Social policy					
Categorical benefits	Unemployment allowance		−0.001	−0.001	−0.002
			(−0.92)	(−0.44)	(−0.94)
	Family allowance		−0.002	−0.003	−0.002
			(−1.55)	(−1.45)	(−1.58)
Pro-poor benefits	Social allowance		−0.01*	−0.005*	−0.008*
			(−2.56)	(−2.27)	(−2.22)
	Housing allowance		−0.000	0.004	0.001
			(−0.85)	(1.56)	(0.23)
*N* (C = 30)		283,078	283,078	47,640	56,635
Intra-class correlation		0121	0097	0102	0098
R2		0107	0107	0093	0103
Bayes information criterium		72,800	72,806	19,662	17,831
Log-likelihood		−36,262	−36,258	−9707	−8790

Note: Source: EU SILC. Displayed are average marginal effects on having low FAH. Sample: all EU SILC countries (C = 30) except for Croatia and Cyprus. Controlled for but not displayed: population density. Significance levels: ^o^p/z > 0.1 (insignificant); +p/z < 0.1; *p/z < 0.05; **p/z < 0.001, Bonferroni significance levels (if different) follow after the slash (/). Z-statistics are shown brackets. Social policy variables were added step-wise, the statistics displayed refer to the model with the highest log-likelihood

#### Individual level influence

In M1, women are showing slightly lower FAH than men, even when controlling for health and chronic illnesses. Furthermore, the influence of household composition is visible in the case of single parent households. These are on average more likely to show low FAH than families with two adults and no children. Also migrant status as well as low and medium education, which capture knowledge of healthcare policies, are significant determinants of limited FAH. Next to these mostly ascriptive characteristics, general cost factors are considered. Urbanisation as well as accessibility of public transport and healthcare control the indirect costs of accessing healthcare. FAH seems to be more difficult in urban than in medium or thinly populated areas, while accessibility of healthcare centres is worse in rural areas. When looking at the socio-economic status, in the full model the household income is most decisive for FAH, followed by unemployment and debt issues.

In Model 3 and 4, the first and second income quintiles are portrayed respectively. There are some changes to the general model. Firstly, individuals with low FAH are characterised by a worse health status and are more likely to have chronic illnesses if they are from the poorest quintiles. Also the risk of low FAH among migrants is elevated if they are from the poorest quintile, while interestingly in the lower middle class migrant status does not show an effect on FAH. Breaking the results down by income quintile also reveals that educational attainment has a significantly higher marginal effect on FAH among individuals from the lowest income quintile, compared to the lower-middle income quintile. The importance of inter-related risk factors for FAH is also portrayed in the variable public transport, which is significant only for the poorest. A last point concerns the impact of debt on FAH. While in the general model its importance lags behind income and unemployment, in the model for the lower middle class being evicted for financial reasons is the key decisive variable.

#### Macro-level influence

Now, the analysis will turn to the macro-level results. The model before adding explanatory variables (not shown) displays an inter-class correlation of 0.25. This means that a quarter of the risk distribution can be assigned to national policies. In M1, the healthcare system variables explain about a half (0.13) of these cross-country differences in FAH. Private out-of-pocket expenditure (OPP) and the physician density are both significant macro determinant of FAH.

In M2, the social policy variables are added to determine the possible impact of transfers for the population as a whole. While in Table 3 they are portrayed inside one model, they were added step-wise to prevent multicollinearity between the variables. When social policies are added, physician density remains significant and OPP drop from a 5 % to a 10 % significance level. At the same time the inter-class correlation (ICC) lowers by 0.02, implying that social policies are able to explain another share of country differences in FAH. Looking at the social policies by category, only one of the pro-poor benefits, namely social allowance is significant. Housing, family and unemployment allowance do not show a significant effect, even though the direction of their effects is negative as expected. The effect of social allowance on FAH is strong enough to be visible in the full model for the whole population, even though it is the least generous of all benefits. However, its marginal effect is low, meaning that social allowance improves the uptake of healthcare services only to a small extent.

When looking at the M3 and M4, the same social policies stay (in-)significant. It is not possible to detect a higher impact of the family and unemployment benefits on the lower-middle income group. Neither are housing benefits significant for the lowest income quintile. It seems rather that social allowance is able to improve FAH for both Q1 and Q2.

## Discussion

In the discussion, the paper will consider the implications of the results obtained, examine the validity of the hypotheses and reflect the limitations of the study.

### Discussion of individual-level factors

Firstly, *direct and indirect costs*: Urbanisation as well as accessibility of public transport and primary care revealed the importance of indirect costs on individuals’ decision to seek care. Transport, while not being significant in the general model, shows significance for the poorest quintile. Also access to primary care shows a higher coefficient for Q1. This confirms the theory set out above that indirect cost factors are particularly important for the poorest income quintile. The second income quintile is suffering more from direct costs, in particular with issues of non-coverage and payment difficulties following unemployment or debt problems. This is visible from the importance of debts and consequential eviction on FAH for Q2, which is the main risk factor linked to socio-economic status. These trends support *H1* set out above. In general, debts and consequential eviction seem to be a factors which has been largely ignored when talking about FAH. Only in recent years, interest in the topic has increased, largely due to increasing debt burdens in the financial crisis and widening knowledge on its mental health implications.

Secondly, *knowledge of the healthcare system*: Healthcare system knowledge showed its importance in particular when combined with low income. The marginal effect of low education is likely to be even underestimated, as knowledge of an illness is a predisposing factor [10] for low FAH to be reported. Individuals have to become firstly aware of their medical issue (e.g. by going to screenings) and perceive it as a problem, before FAH arises. Individuals with low health knowledge may thus report low unmet medical needs which do not correspond to the number of untreated health issues they are facing. For migrants, next to low knowledge of the healthcare system, also discrimination can be an important reason for low FAH, as has been shown by the Eurofound report [7]. In addition, the lack of information of healthcare services providers about migrant’s rights might be an additional point which leads to low FAH. The shift of universal health system in the crisis to restrict their services based on citizenship will have likely improved this risk factor.

Thirdly, the results show the role of *intra-household need prioritisation* [48]. The impact of need prioritisation is shown in the low FAH of women and single parent households, which are more prone to forgo medical treatments. Given same monetary and health needs, their decision not to seek care is probably taken in order to save income for their children’s needs. These individuals are a risk group, which has not been addressed in prior analysis, as they will not voice their hardship.

### Discussion of macro-level factors

Firstly, *healthcare policies:* The significance of healthcare system characteristics and in particular of healthcare financing and availability was expected following Gelormino et al. [34]. From M1, it is deductible that increasing the pure density and number of primary care services will improve the financial accessibility of medical care. Likewise lowering the private health expenditures by public provision of government supported or free services, treatments and pharmaceuticals would be a way of improving FAH from the medical side, corresponding to what has been proposed by proposed by Rezayatmand et al. [49].

Secondly, *social protection policies*: In M2, the idea is confirmed that healthcare financing and social policies are two sides of the same coin termed healthcare uptake, as the significance of healthcare financing drops when social policies are considered at the same time. Next to healthcare system variables, social allowance is showing importance in improving FAH. This implies that *H2* can be confirmed, social protection policies are able to improve the household income of the poorest and are efficient in lowering unmet medical needs for financial reasons. The quintile-specific importance of social protection policies can however not be affirmed as expected. Social allowance ameliorates FAH for the first and second income quintile alike. While *H3a* can thus be confirmed, *H3b* has to be rejected. Social allowance seems to be having a double dividend. In the first place, it is directed at the Q1; with its basic income provision it cushions health expenses for this group. In the second place, it seems to be additionally bringing about a threshold effect, lifting wages and income for the lower-middle income class (by increasing their reservation wage). The validity of the results obtained seems high, as the impact of social allowances was confirmed when using Eurostat expenditure data instead of received benefits.

### Limitations of the study

The limitations of the study stem from the EU-SILC income data and the subjectivity of responses to the FAH questions. The EU-SILC can give rise to certain difficulties in the comparability of results between countries due to variations in the population sampling, the survey methods and the imputation method for non-response [38]. The unmet medical needs question which forms the basis of the analysis will be consistently interview-based, so no differences in comparability should arise (given that an adequate translation guarantees the equivalence of questions). The comparability of household income variables (and their components) with actual living conditions at the time of the interview (*t*), and among countries, is more questionable. Given that the past year (*t*-1) forms the reference period for income, this may lead to a discrepancy with related variables. Depending on whether an increase or decrease of income occurred over the course of the year, the impact of income on FAH might be over- or understated. Moreover, net income data is not available for all income components in all countries. This impacts the representativeness of the housing, family, social assistance and unemployment benefit data which have to rely on gross income supplements (different tax regimes might lead to changes in cross-country rankings for net data). As long as the unmet medical need questions is not covered in the longitudinal survey, cross-country comparisons will however form the best method to evaluate the impact of social policies.

Next to data concerns, the objectivity of variables collected by personal interviews might be questioned. A draw-back from the operationalization of FAH is that “unmet medical need” is self-reported. This implies that individuals have to become aware of their medical issue (e.g. by going to screenings) and perceive it as a problem, before an unmet medical need can arise [10]. Individuals with low health knowledge may thus underreport unmet medical need. As low income and low education are often statistically correlated, this might lead to an underreporting bias in particular in the Q1. To counter this bias, FAH is calculated for different population groups while holding health and chronic diseases constant. The subjective nature of the FAH variable, is accentuated by using “enforced unmet medical needs”, which includes waiting lists and transport costs in addition to the pure economic costs of visiting a doctor. While this approach was adopted (similar to [16], and supported by findings of [50]), in order to reflect the multiple risk factors linked to low income, the subjective nature of the waiting time variable (which could be measured in reference to purely public healthcare waiting time or by assessing the relation between public and private provision or even include the complex issue of bribe) [50] warrants for further vagueness. Given, however, that unmet medical needs for financial reasons are often underreported due to non-awareness and the stigma involved in admitting such financial hardship, the broader approach is likely to expose a more realistic number of cases with FAH.

## Conclusion

The article set out to depict financial accessibility to healthcare (FAH) for low income groups at a moment of high healthcare demand and restricted supply. The purpose of this study was to analyse how aspects of social policy which increase the purchasing power of households may mitigate the effects of the crisis on the ability of integrated health systems to meet need for care. The article differs from previous research by setting the focus on the demand side of individuals which is pre-structured by social policies. The results show clearly that households under financial stress are likely to be deterred from accessing healthcare due to direct and indirect costs implied. Among low-income households those with debt issues show the most elevated risk of low FAH, followed by those being at the same time unemployed or under-educated. Another risk group which is often not discussed are single-parent families with low income. It is important to facilitate access for these groups as low FAH may potentially worsen people’s chances in the labour market in the long run. Moreover, when health services are used by low-income individuals despite the financial burden implied, resources are averted from other important elements of a household’s budget, e.g. food and rent payments [17], thereby increasing the risk of recurring illnesses, leading into a poverty trap.

The multi-level analysis revealed that social allowance policies contribute meaningfully to the accessibility of the healthcare system thereby complementing healthcare services in the task of ensuring a healthy population. Besides generally improving purchasing power of low-income households by social allowance, policies should address households who have experienced debt and eviction for financial reasons. Social policy-makers should take this group into consideration and provide these highly vulnerable individuals with free access to healthcare. While this article examined the financial side to accessing healthcare only, the ability to seek, reach and engage in healthcare [10] is equally important. Measures aimed at explaining the functioning of the healthcare system and healthcare rights to migrants and at facilitating interactions with healthcare professionals for individuals with few health knowledge, will be equally valuable for improving access of vulnerable groups.

In sum, we can conclude that – contrary to the effect budgeting hypothesis – low-income groups make smart choices, even in times of recession, by using higher purchasing power for accessing healthcare, thereby improving population health. We can deduce that a re-discovery of the values of automatic stabilizers is urgent. Seemingly simple cost-containing policy solutions may incur higher costs in the long run and worsen population health.

## Abbreviations

CEEC: Central and Eastern European countries
EU: European Union
FAH: Financial accessibility of healthcare
GP: General practitioner
ICC: Intra-class correlation (measure in multi-level analysis)
OECD: Organisation for Economic Co-operation and Development
OPP: Out-of pocket expenditure
Q1: First income quintile (the lowest 20 % of the national income distribution)
Q2: Second income quintile (the second lowest 20 % of the national income distribution)
WHO: World Health Organisation

## Footnotes

1 In this paper, households in the lowest income quintile (Q1) are referred to as “income poor”. This concept is broader than the official EU definition of “at risk of poverty”, which takes 60 % of the national median, and according to which just the lowest 16.8 % of the European population were poor in 2012 (national values varying between 7.9 in Iceland and 23.1 in Greece). Households in the second lowest income quintile (Q2) are referred to as the “lower middle class”, they are above the national poverty line. National social protection policies can yet again define their target group in a different way (e.g. taking income above the minimum wage).

2 Health spending measures the costs of health services (out/in-patient care, long-term care, prevention and public health services) and goods (pharmaceuticals and other medical goods) [51].

3 User fees can take the form of ‘co-payments’ (users pay a fixed amount per doctor visit), ‘co-insurance’ (users pay a proportion of total medical costs) or ‘deductible pricing’ (users bear costs until a fixed amount). Co-payments are the most common way of patient-cost-sharing in Europe [31].

4 For countries subject to bailout agreements the healthcare sector reforms were in part mandated by the troika comprising the European Commission, the European Central Bank and the International Monetary Fund [52]. For other European countries, increased healthcare efficiency was recommended in the European Semester process [53].

5 During the Great Recession, 17 out of the 28 European Member States lowered their public health spending as a percentage of GDP [14]. Cuts were applied to healthcare expenditures (e.g. by lowering wages for staff and re-setting prices paid for generic drugs) and by changing the structural determinants for access to healthcare: Infrastructure was reduced by lowering the number of hospital beds, by closing hospitals in rural areas (e.g. in Sweden, Bulgaria and Romania) [7, 33], and by laying off staff [32]. Population coverage was made more restrictive by shifting from universal service provision to provision based on citizenship (Spain). In other countries, long-term unemployed (Greece) and individuals with private debts towards public institutions (Slovenia) faced difficulties in insurance coverage [7]. Service coverage was reduced by lowering publicly provided support for purchasing pharmaceutical products and by cutting treatment programmes for mental health, physiotherapy and non-urgent ambulance services [32]. In Greece, Ireland, Italy, Latvia, Portugal, Romania, the Slovak Republic and Slovenia co-payments were introduced or raised, increasing private healthcare costs by up to 15 % [7, 14].

6 This minimum social standard is often based on a basket of goods, whose size and composition is highly dependent on the welfare state regime and a country’s living standard [54].

## References

[1] Bradley EH, Elkins BR, Herrin J, Elbel B (2011). Health and social services expenditures: associations with health outcomes. BMJ Qual Saf.

[2] Lundberg O, Åberg Yngwe M, Kölegård Stjärne M (2008). The role of welfare state principles and generosity in social policy programmes for public health: an international comparative study. Lancet.

[3] Figueras J, McKee M (2012). Health systems, health, wealth and societal well-being: assessing the case for investing in health systems. European observatory on health systems and policies series.

[4] WHO Europe (2013). Health, health systems and economic crisis in Europe. Impact and policy implications.

[5] Thomson S, Foubister T, Mossialos E (2010). Can user charges make health care more efficient?. Br Med J.

[6] Kiernan, F. What Price Austerity – A nation’s health? The effect of austerity on access to health care in Ireland. The European Journal of Public Health. 2014; http://eurpub.oxfordjournals.org/content/24/suppl_2/cku165.110.

[7] Eurofound (2014). Access to healthcare in times of crisis.

[8] Jacobs M, Ir P, Bigdeli M (2012). Addressing access barriers to health services: an analytical framework for selecting appropriate interventions in low-income Asian countries. Health Pol Plann.

[9] Bigdeli M, Jacobs B, Tomson G (2013). Access to medicines from a health system perspective. Health Pol Plann.

[10] Levesque JF, Harris MF, Russell G (2013). Patient-centred access to health care: conceptualising access at the interface of health systems and populations. Int J Equity Health.

[11] Wendt C (2009). Mapping European healthcare systems: a comparative analysis of financing, service provision and access to healthcare. J Eur Soc Pol.

[12] Reibling N (2010). Healthcare systems in Europe: towards an incorporation of patient access. J Eur Soc Pol.

[13] Peters DH, Garg A, Bloom G (2008). Poverty and access to health care in developing countries. Ann N Y Acad Sci.

[14] OECD (2014). Health at a glance 2014. OECD indicators.

[15] Penchansky R, Thomas JW (1981). The concept of access: definition and relationship to consumer satisfaction. Med Care.

[16] Kyriopoulos II, Zavras D, Skroumpelos A, Mylona K, Athanasakis K, Kyriopoulos J (2014). Barriers in access to healthcare services for chronic patients in times of austerity: an empirical approach in Greece. Int J Equity Health.

[17] Kutzin J (2000). Towards universal health care coverage. A goal-oriented framework for policy analysis. Health, Nutrition and Population (HNP) discussion paper.

[18] Van Doorslaer E, Masseria C, Koolman X (2006). Inequalities in access to medical care by income in developed countries. Can Med Assoc J.

[19] Mielck A, Kiess R, Kneseback O, Kunst A, Mackenbach JP (2007). Association between access to health care and household income among the elderly in 10 western European countries. Tackling health inequalities in Europe: an integrated approach. Final report.

[20] Hart JT (1971). The inverse care law. Lancet.

[21] Dahlgren G, Whitehead M (1992). Policies and strategies to promote equity in health.

[22] Stuckler D, Basu S (2013). The body economic. Why austerity kills.

[23] Mladovsky P, Ingleby D, McKee M, Rechel B (2012). Good practices in migrant health: the European experience. Clin Med.

[24] Eurofound (2013). Impacts of the crisis on access to healthcare services in the EU.

[25] Albrecht M, Otgeton S, and Ochmann RR. Faktencheck Gesundheit. Regionale Verteilung von Arztsitzen (Ärztedichte). Gütersloh: Bertelsmann Stiftung; 2014

[26] Moran M (2000). Understanding the welfare state: the case of health care. British Journal of Politics and International Relations.

[27] Bambra C (2005). Cash versus services: ‘worlds of welfare’ and the decommodification of cash benefits and health care services. J Soc Pol.

[28] Montanari I, Nelson K (2013). Social service decline and convergence: how does healthcare fare?. J Eur Soc Pol.

[29] Mladovsky P, Srivastava D, Cylus J, Karanikolos M, Evetovits T, Thomson S (2012). Health policy in the financial crisis. Eurohealth.

[30] Hacker JS (2004). Privatizing risk without privatizing the welfare state: the hidden politics of social policy retrenchment in the United States. Am Polit Sci Rev.

[31] Tambor M, Pavlova M, Woch P, Groot W (2010). Diversity and dynamics of patient cost-sharing for physicians’ and hospital services in the 27 European Union countries. Eur J Pub Health.

[32] WHO Europe (2012). Health policy responses to the financial crisis in Europe.

[33] Karanikolos M, Mladovsky P, Cylus J, Thomson S, Basu S, Stuckler D (2013). Financial crisis, austerity, and health in Europe. Lancet.

[34] Gelormino E, Bambra C, Spadea T, Kunst A, Bellini S, Costa G, Mackenbach JP (2007). The effects of health care reforms on health inequalities: a review and analysis of the European evidence base. Tackling health inequalities in Europe: an integrated approach. EUROTHINE final report.

[35] Whiteford P, OECD (2008). How much redistribution do governments achieve? The role of cash transfers and household taxes. Growing unequal?.

[36] Immervoll H (2009). Minimum-income benefits in OECD countries: policy design, effectiveness and challenges. IZA discussion paper No. 4627.

[37] Scheiwe K, Eekelaar J, George R (2014). State support for families in Europe: a comparative overview. Routledge handbook of family law and policy.

[38] Iacovou M, Kaminska O, Levy H. Using EU-SILC data for cross-national analysis: strengths, problems and recommendations. ISER Working Paper Series. 2012; 03

[39] Rabe-Hesketh S, Skrondal A (2008). Multilevel and longitudinal modeling using Stata.

[40] Hox JJ (2010). Multilevel analysis: techniques and applications.

[41] Burgard S, Kalousova L (2015). Effects of the great recession: health and well-being. Annu Rev Sociol.

[42] Bryan ML, Jenkins SP. Regression analysis of country effects using multilevel data: A cautionary tale. ISER Working Paper Series. 2013; 14

[43] Austin PC (2010). Estimating multilevel logistic regression models when the number of clusters is low: a comparison of different statistical software procedures. Int J Biostat.

[44] Goldman M. Why is multiple testing a problem? Statistics for Bioinformatics; 2008, Stat C141

[45] Bartus T (2005). Estimation of marginal effects using margeff. Stata J.

[46] Mood C (2010). Logistic regression: why we cannot do what we think we can do and what we can do about it. Eur Sociol Rev.

[47] Best H, Wolf C (2012). Modellvergleich und Ergebnisinterpretation in Logit- und Probit- Regressionen. Kölner Zeitschrift für Soziologie.

[48] Gabos A, Ozdemir E, Ward T (2011). Material deprivation among children. Social situation observatory – income distribution and living conditions 2011. Research note 7/2011.

[49] Rezayatmand R, Pavlova M, Groot W (2012). The impact of out-of-pocket payments on prevention and health-related lifestyle: a systematic literature review. Eur J Pub Health.

[50] Kentikelenis A, Karanikolos M, Papanicolas I (2011). Health effects of financial crisis: omens of a Greek tragedy. Lancet.

[51] OECD. Health spending (indicator). 2016. doi: 10.1787/8643de7e-en. Retrieved from https://data.oecd.org/healthres/health-spending.htm. (Accessed on 03 March 2016)

[52] Fahy N (2012). Who is shaping the future of European health systems?. Br Med J.

[53] Azzopardi-Muscat N, Clemens T, Stoner D, Brand H (2015). EU Country Specific Recommendations for health systems in the European Semester process: Trends, discourse and predictors. Health Policy.

[54] Nelson K (2012). Counteracting material deprivation: the role of social assistance in Europe. J Eur Soc Pol.

